# Exploring the need for lower limb prosthetic guidelines in South Africa’s private healthcare sector

**DOI:** 10.33137/cpoj.v7i2.44450

**Published:** 2025-02-12

**Authors:** B Theron, S Visagie

**Affiliations:** University of Stellenbosch, Division of Disability and Rehabilitation Studies, Faculty of Medicine and Health Sciences, South Africa.

**Keywords:** Prosthetist, User Experience, Prosthesis, Funding Policy, Rehabilitation, Lower Limb Amputation, South Africa, Prosthetic Guidelines

## Abstract

**BACKGROUND:**

Evidence based guidelines can assist with prosthetic component selection and clinical intervention. There is limited evidence on lower limb prosthetic prescription guidelines in the South African private health care sector.

**OBJECTIVE:**

To explore the need for lower limb prosthetic prescription guidelines in the South African private healthcare sector.

**METHODOLOGY:**

Three main funders of lower limb prosthetics in the South African private healthcare sector (Road Accident Fund (RAF), Workmen’s Compensation Fund (WCA), and Council of Medical Schemes (CMS)) were explored using a case study design. Data were collected from six regulatory documents, sixteen purposively sampled prosthetic users, who received services from these funders, and seven key informants. Documents were assessed with the Appraisal of Guidelines for Research & Evaluation II (AGREE II), across six domains. Data from users and key informants were collected with telephonic, semi-structured interviews guided by interview schedules. Interview schedules were self-developed and tailored for each participant group. AGREE II data were analyzed descriptively. Inductive thematic analysis was used for interview data.

**FINDINGS:**

Across cases, the “Scope and Purpose” domain scored the highest: 50% (WCA), 47% (CMS), and 22% (RAF). “Editorial Independence” scored 0% for all three cases. Other challenging domains were “Applicability” (WCA: 17%, CMS: 6%, RAF: 6%) and “Rigour of Development” (WCA: 8%, CMS: 25%, RAF: 0%). The following three cross-case themes emerged from the interviews: “Guideline Availability and Necessity” showed that guidelines were seldom used and that guidelines could be beneficial; “Purpose of a Lower Limb Prosthetic Guideline” indicated that guidelines can support accessible, equitable, ethical, and transparent services; and “Guideline Development Requirements” explained that an evidence based collaborative process, facilitated by an independent body should underscore guideline development.

**CONCLUSION:**

Evidence based, standardized, transparent guidelines will be beneficial to direct prosthetic service delivery in the South African private healthcare sector. The guidelines must be applicable, rigorously developed, and show editorial independence.

## INTRODUCTION

Lower limb amputations and prosthetic devices have been around for ages. Over time, prosthetic materials and manufacturing techniques have developed from hand tooled wood and leather to metal, space age materials and computer aided design and manufacturing. These advances allow the manufacturing of prostheses that can restore user functionality to before or even above pre-amputation levels.^1^ However, more advanced materials are expensive and are often not used in lower- and middle-income countries, including South Africa.^2^

According to a 2022 national census there are around 430,000 upper and lower limb prosthetic users in South Africa.^3^ The average age of persons living with an amputation in Africa and South Africa is lower than in developed countries and often younger than 60 years of age.^4^ Thus, the functional requirements of occupations must be considered when prosthetic components are selected.^5^ Lower limb prosthetic services are mainly funded by one of four sources in South Africa, namely public and private healthcare funding, as well as road, and work-related accident funding.

Successful fitting of lower limb prostheses is dependent on optimal prosthetic component prescription, based on a comprehensive assessment of physical needs, life roles, and the environment in which the person is to function.^6,7^ The componentry must match the users' abilities, goals, and daily use requirements.^6^ Lower limb prosthetic componentry which accommodates activity level and user need are often expensive with funding not readily available in developing markets. However, cost saving should not be the primary focus when prescribing prosthetic componentry.^7,8^ A high initial financial investment increase Quality Adjusted Life Years^9^ and decrease future financial expenditure due to decreased secondary complications and increased product lifespan.^10^ Thus, higher initial fiscal investment should not be seen in isolation as this investment has far-reaching positive effects on socio-economic and healthcare environments.^9,10^ Clinical practice guidelines can assist with prescribing appropriate prosthetic components in accordance to user needs.^8^

Clinical practice guidelines provide evidence-based recommendations intended to optimise patient care while considering the benefits and harms of alternative options.^11^ Guidelines provide justification for interventions such as prosthetic component selection with transparency to all parties involved.^8^ Comprehensive, effectively implemented guidelines can enhance quality and consistency of care.^12^ A lack of or inferior guidelines can cause gaps in service delivery that can disadvantage the end user.^13^

Healthcare funders, prosthetists, rehabilitation specialists and prosthetic users must be involved in the development of lower limb prosthetic provisioning guidelines, as broad stakeholder involvement allows for transparent guideline formation beneficial to all parties.^8,14^ The quality of guidelines must be ensured through transparency and rigour during development. Central to the process is the intended human activity based on functions and anatomical characteristics.^8^ Empirical knowledge is essential. Local medical device regulations should also be considered. Lower limb prosthetic guidelines have been adopted in various sectors in different countries.^6,8,15^ However, information on the effect of lower limb prostheses guideline implementation is limited.^15^

Anecdotal information suggests little national and/or in-house guidelines to guide prosthetic service delivery in South Africa. Usually, componentry is prescribed and funded based on the user's activity level as determined by the Amputee Mobility Predictor with/without Prosthesis (AMPPRO/AMPnoPRO).^16^ Furthermore, Certified Prosthetists and Orthotists (CPOs) clinical expertise and experience guides prosthetic prescription. Clinical knowledge plays an important role in appropriate prescription but must be supported by evidence.^6,8^ Not including evidence in prescription decisions may lead to deviation in prosthetic prescription as well as over-or under treatment. These challenges prompted this study with the aim to explore and describe the need for a lower limb prosthetic prescription guideline in the private healthcare sector in South Africa.

### Conceptual framework

**Figure 1:**
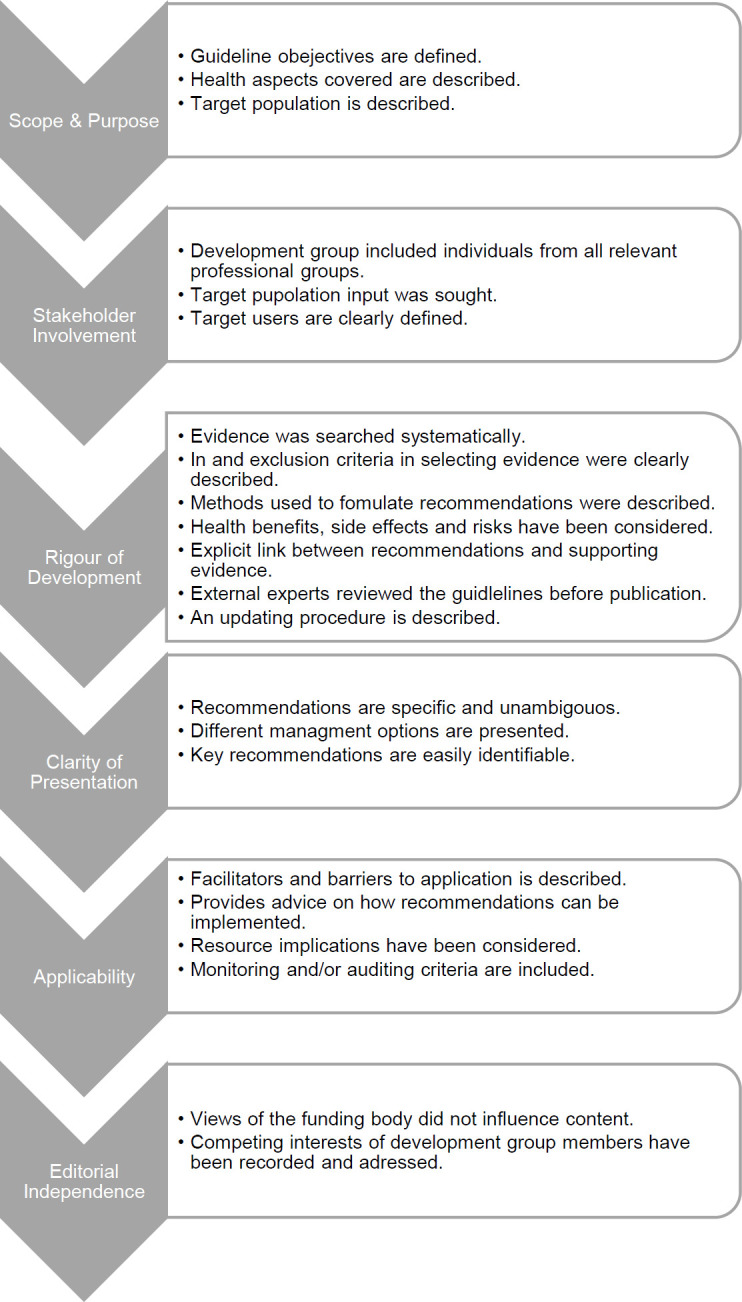
The six domains and domain items of the AGREE II (adapted from Brouwers et al.^^14^^).

The updated Appraisal of Guidelines for Research and Evaluation (AGREE II) provided a framework for the study as well as a document appraisal tool (**Figure 1**).^14^ The AGREE II framework can be used to develop guidelines and evaluate the quality of guidelines. Geertzen et al.^8^ successfully used the AGREE II to formulate a lower limb prescription guideline in the Netherlands.

## METHODOLOGY

**Figure 2:**
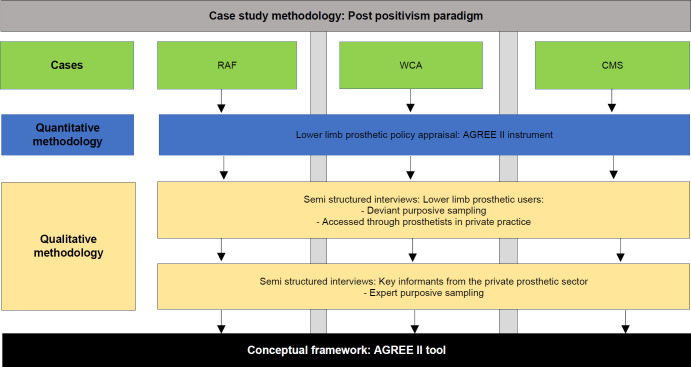
An illustration of case study methodology through a mixed method approach used in the study.

An exploratory case study methodology was used because it provides insight and facilitates understanding of complex phenomena within a defined context. It can explore how similar issues are dealt with in different contexts and how context influences the phenomenon being researched.^17^ Qualitative and qualitative data were collected as illustrated in **Figure 2**.

The three cases that were explored in the study are the main funders of lower limb prosthetics in the South African private healthcare sector:

- Road Accident Fund (RAF)
- Workmen's Compensation Fund (WCA)
- Council of Medical Schemes (CMS)

**RAF**: The RAF (**Table 1**) is a social insurance service, funded through a national fuel levy, and provides compulsory cover to all road users in South Africa. Once liability has been accepted, the RAF shall “*compensate for costs of the future accommodation of any person in a hospital or nursing home or treatment of or rendering of a service or supplying of goods*.”^18^ Goods and services include lower limb prosthetic services. Coverage is provided for the remainder of the beneficiary's life. For the first time, the RAF published a product list for reimbursement in 2022.^19^

**Table 1:** Overview of lower limb prosthetic funders in South Africa.

	RAF	WCA	CMS	Public Health Sector
**Reimbursement system**	Medical schemes professional fee tariff list + NAPPI codes (medicine, consumables and devices)	Annual published Government Gazette	Medical schemes professional fee tariff list + NAPPI codes (medicine, consumables and devices)	Subsidised by government with patient co-payment based on income*
**Responsible department**	Department of Transport	Department of Employment and Labour	Department of Health	Department of Health
**Percentage of population eligible for coverage**	100%	67% (aged between 15-64)**	15%***	100%
**Funding method**	Fuel levy	Compulsory deduction from salary or wage	Medical Schemes via members private monthly contributions	National Health Budget

* https://www.westerncape.gov.za/general-publication/western-cape-government-hospital-tariffs-overview?toc_page=3

** https://census.statssa.gov.za/#/

*** https://www.medicalschemes.co.za/preliminary-industry-trends/

**WCA**: The WCA (**Table 1**) administers the Compensation for Occupational Injuries and Diseases Act No 130/1993^20^ as amended by the Compensation for Occupational Injuries and Diseases Act No 61/1997.^21^ The Act provides compensation for disablement or death caused by occupational injuries or diseases. Employees who sustained a lower limb amputation due to a work-related injury, are automatically covered for life once liability has been accepted. An annually published gazette provides guidance on prosthetic prescription, eligibility criteria, renewal periods, application forms and reimbursable professional fees and products.^16^ The 2023 gazette included technologically advanced prosthetic componentry for prescription.^16^

**CMS**: The CMS (**Table 1**) is a statutory body whose operational objectives are described in the Medical Schemes Act (Act 131 of 1998).^22^ The CMS regulates and monitors the functioning of medical schemes in South Africa. Lower limb prosthetics are covered under Prescribed Minimum Benefits (PMB), the minimal level of care that the medical scheme is obliged to fund without copayments or deductibles. Prosthetics should be funded in the private sector at least equal to what is provisioned for in the public sector.^23,24^ CMS has published a guideline document on amputations (non-specific) which outlines the basic coverage that medical schemes should offer to their members.^25^

The Public healthcare sector was excluded from the study as the purchasing methods and service delivery mechanisms differ in each of the nine provinces in South Africa and differ from methods used in the private sector. The three private funders have a homogenic approach to funding lower limb prostheses. The Public healthcare sector receives funding from the National Healthcare budget of the Department of Health (DoH) which provides the general public with access to prosthetic care (although basic). The DoH has various orthotics and prosthetic facilities in each province where CPO's are employed by DoH to manufacture and fit prosthetic devices. These facilities are fully financially dependent on DoH for (but not limited to): infrastructure, facility maintenance, human resources, orthotic and prosthetic components and consumables, as well as general running costs of an orthotic and prosthetic facility.

### Data sources

Data were collected from documents, key informants, and lower limb prosthetic users. Documents were retrieved from the public domain, as a formal request for lower limb prosthetic prescription guidelines, regulatory frameworks, and/or policies to the three entities yielded no response.

Seven key informants were purposefully sampled using expert purposive sampling.^26^ They included management level employees from the three cases, as well as individuals from educational institutions, professional bodies, and prosthetic component suppliers. The key informants have knowledge and experience working in the sector and could give insight into current practices and the need (or not) for a lower limb prosthetic prescription guideline. The contact details of these individuals are available in the public domain. They were contacted via phone by the researcher. The aim of the study and their role were explained, also that the interview would be audio recorded for accurate transcriptions. After willingness to participate in the study was expressed, a study information leaflet and consent form for virtual data collection were sent via email. Once the signed informed consent was received, a telephonic interview was scheduled.

Sixteen lower limb prosthetic users, whose prostheses were funded by one of the three cases, were sampled through maximum variation purposive sampling. The study population of lower limb prosthetic users was unknown and could not be accessed through a database. CPO's working in the private healthcare sector in various geographical regions of South Africa were contacted and requested to reach out to possible lower limb prosthetic users to participate in the study. Twenty-one possible participants were identified by CPOs based on the inclusion and exclusion criteria. CPOs asked verbal consent to share their contact information with the researcher.

***Prosthetic user inclusion criteria:***

- Persons with major lower limb amputation/s (unilateral or bilateral).
- Persons who received a lower limb prosthesis at least six months before commencing data collection.
- Insured with the RAF, WCA or a private medical scheme registered with the CMS.
- Ability to communicate in any of the official languages in South Africa.

***Prosthetic user exclusion criteria:***

- Lower limb prosthetic users who had stroke, spinal cord injury, or traumatic brain injury.
- Persons who could not participate in a virtual or telephonic interview due to lack of access to necessary hardware or speech impairments.
- Persons who could not provide informed consent due to cognitive impairments.

The researcher contacted all twenty-one possible participants by telephone; five did not respond. The study was explained to them, and the provisional consent for their participation in the study was obtained. The study's information leaflet and consent form for prosthetic users were sent to participants via email.

Sixteen individuals completed the consent form and sent it back to the researcher, whereafter an appointment for an audio recorded interview was made. Lower limb prosthetic users who were both satisfied and dissatisfied with their prosthesis were included in this study.^26^ To further ensure variation, prosthetic users were sampled to include all sexes, age groups, major lower limb amputation levels and urban and rural lower limb prosthetic users. Data saturation was reached after sixteen interviews. Although lower limb prosthetic users of only six out of the nine provinces in South Africa were interviewed, the cohort included both urban and rural lower limb prosthetic users. The three cases studied operate the same across all provinces thus the experiences of lower limb prosthetic users would be similar.

As the study was novel, there was no available questions which could have been accessed or informed the interview schedule. The interview questions were formulated based on the AGREE II tools six domains and researchers experience in the industry. All interview schedules were in English, and interviews were conducted in both English and Afrikaans (although any official South African language could have been accommodated if the need had arisen).

### Data collection

The AGREE II reporting checklist was used to appraise documents. The AGREE II consists of 23 items in 6 domains, scored on a scale from 1 (strongly disagree) to 7 (strongly agree). Domain scores are calculated by summing item scores and scaling the total as a percentage of the maximum score.^27^ Higher AGREE II scores indicate higher quality guidelines. A guideline can be “strongly recommended” if four (or more) out of the six domain scores are above 60%.^28^ Guidelines can be “*recommended with provisions or alterations*” if at least two out of the six domains scored between 30%-60%. Items of the AGREE II are valid and useful. The tool is “*appropriate, easy to use, and helpful in differentiating guidelines of varying quality*”.^29^ The two authors appraised the documents individually after which domain averages were calculated.

Qualitative data were collected through semi-structured, telephonic interviews, by the first author between October 2022 and April 2023. Two interview schedules, informed by the AGREE II, were developed for the key informants and users respectively.

***Prosthetic user questions:***

- Can you please describe the process of getting your current prosthesis?
- What information was available to help you understand the process?
- To what extent was your opinion on the type of prosthesis and components considered?
- What could your funder have done differently in this process?
- What advice do you have for other new lower limb amputees in accessing prosthetic care?

***Key informant questions:***

- What challenges do we experience in lower limb prosthetic prescription and provision in the South African private healthcare sector?
- What role can lower limb prosthetic guidelines play in prosthetic prescription in the South African private healthcare sector?
- What would the ideal situation be in the prescription of lower limb prosthetics in the private healthcare market?

### Data analysis

Data were analyzed for each case individually after they were integrated. Data from the AGREE II reporting checklist were collated and summarised. Inductive thematic analysis was used to analyze qualitative data. After coding, provisional themes were developed separately by the authors. Consensus was reached and themes were named and defined.

### Rigour

Case study methodology allows different facets and views of a phenomenon to be explored, through multiple cases and in-depth analysis of multiple information sources to increase the credibility of the study.^17^ Credibility was further enhanced through purposive sampling and data saturation.^30^ Transferability was supported by describing the cases and providing demographic information on participants.^17^ Confirmability was supported through triangulation and researcher reflexivity.^30^ Dependability was sought through triangulation, researcher reflexivity and the presentation of limitations.^30^

### Ethical considerations

Ethical approval was obtained from the health research ethics committee at Stellenbosch University (N22/01/002). Participation in the study was voluntary. The informed consent form was sent to participants electronically for them to sign and return before the interview was scheduled.^31^

## RESULTS

### Document analysis

The following documents were assessed:

- Road Accident Fund Act (Act 56 of 1996),^18^ and Road accident fund medical tariff (19 August 2022, No 46747).^19^
- The Compensation for Occupational Injuries and Disease (COID) Act (Act 130 of 1993)^20^ and the annually published Government Gazette (Volume 693, March 2023. No 48299).^16^
- The Medical Schemes Act, (Act 131 of 1998)^22^ and the CMScript - Amputations (issue 2 of 2023).^25^

The highest score overall was 50% (WCA, Scope and Purpose). All three cases scored 0% for Editorial Independence. Two WCA domains, Scope and Purpose and the Clarity of Presentation, scored 50% and 44% respectively. Two CMS domains, Scope and Purpose and Stakeholder Involvement, scored 47% and 30%, respectively. RAF scores were below 30% in all domains (**Table 2**).

**Table 2:** AGREE II domain scores.

Domains	RAF score	WCA score	CMS score
1-Scope and Purpose	22%	50%	47%
2-Stakeholder Involvement	11%	22%	30%
3-Rigour of Development	0%	8%	25%
4-Clarity of Presentation	19%	44%	25%
5-Applicability	6%	17%	6%
6-Editorial Independence	0%	0%	0%
**Recommend for Use**	**No**	**With modifications**	**With modifications**

### Demographic information of participants

Prosthetic users ages ranged from 4 to 66 years. In the instance of minors, their mothers were interviewed. The average time since amputation was 12 years (**Table 3**). Key informants were employed between two and fifteen years in their current role, which provided the basis for sampling (**Table 4**).

**Table 3:** Demographic details of user participants.

Patient ID	Sex	Age*	Occupation	Amputation level	Years since amputation	Year of amputation	Reason for amputation	Province
RAFU1	Male	29	Mechanic	Transtibial	5	2018	Motorbike accident	Gauteng
RAFU2	Male	28	Lecturer - Engineering	Trans-femoral	12	2011	Motorbike accident	North West
RAFU3	Female	35	Unemployed	Trans-femoral	4	2019	Pedestrian accident	Western Cape
RAFU4	Male	16	Scholar	Trans-femoral	8	2015	Pedestrian accident	Western Cape
RAFU5	Female	63	Administrator	Transtibial	18	2005	Pedestrian accident	Gauteng
RAFU6	Male	47	Electrician	Knee disarticulation	5	2018	Motorbike accident	Gauteng
WCAU1	Male	53	Unemployed	Transfemoral	8	2015	Work related car accident	Eastern Cape
WCAU2	Male	52	Supervisor - security	Transfemoral	16	2007	Gunshot	Western Cape
WCAU3	Male	59	Unemployed	Transtibial	3	2020	Falling at work	Western Cape
WCAU4	Male	25	Unemployed	Knee disarticulation & Transtibial	5	2018	Aerospace related incident	Gauteng
WCAU5	Male	66	Pensioner	Transtibial	41	1982	Work related car accident	Kwa-zulu Natal
CMSU1	Male	6	Scholar	Bilateral Transtibial	6	2017	Congenital	Gauteng
CMSU2	Female	5	Scholar	Transtibial	5	2018	Congenital/Infection	Gauteng
CMSU3	Male	39	Personal trainer	Transfemoral	1	2022	Infection	Mpumalanga
CMSU4	Male	60	Project manager	Transtibial	4	2019	Diabetic	Mpumalanga
CMSU5	Male	50	Managing Director	Bilateral Transtibial	50	1973	Congenital	Western Cape

* Years

**Table 4:** Demographic details of key informants.

ID	Sex	Age	Type of organization	Role at organisation	Professional qualification	Time at organization*	Province
KI1	Female	42	Social insurer	Acting general medical manager	Medical doctor	4	Gauteng
KI2	Male	50	Regulatory body	Former senior clinical manager	Medical doctor	6	Gauteng
KI3	Male	48	Professional body	Chairman – Professional body & practicing CPO	Orthotist & Prosthetist	3	Gauteng
KI4	Female	42	Social insurer	Chief Director – Rehabilitation & orthotics	Occupational therapist & MBA	4	Gauteng
KI5	Male	34	Prosthetic device supplier	Head of government sales & stakeholder management	Business management	6	Gauteng
KI6	Female	50	Academia	Senior lecturer	Orthotist & Prosthetist	15	Gauteng
KI7	Female	40	Prosthetic device supplier	Medical device supplier - owner	Psychology	2	Gauteng

* Years

### Emerging themes

Three themes with subthemes were identified as shown in **Table 5**.

**Table 5:** Themes and subthemes identified from participant data.

Theme	Subthemes
Guideline Availability and Necessity	
Purpose of a Lower Limb Prosthetic Guideline	A. Guiding clinical prescription responsive to user needs. B. Supporting adequate funding in a timely manner. C. Equity, transparency, and fair re-imbursement. D. Multi-disciplinary rehabilitation. E. Case managers. F. Information sharing. G. A list of preferred practitioners.
Guideline Development Requirements	H. Driven by an independent entity. I. Collaboration and communication. J. Use of available evidence.

### Theme 1: Guideline Availability and Necessity

Key informant (KI2) indicated that a lower limb prosthetic guideline will “*streamline the care and unify the care such that the minimum standard is applicable across all sectors*”. KI3 felt that guidelines, “*make it a lot easier and have a lot less red tape whereby you can still provide your patients with a reasonable prosthesis*”. Key informants also indicated the current insufficiency of guidelines. KI3 explained that there “*isn't norms, and the norms that are there are old and antiqued*”. Although some funders might have guidelines, “*they don't like to share their guidelines, and keep it as their intellectual property*” (KI4). The lack of guidelines was confirmed by users of all three cases. Lengthy procedures and complex requirements were evident.

“*If they can tell you from point A, I want ABCDEF, you can send everything one time…it is very difficult…it is very complicated*” (CMSU2).

“*Beyond reason, takes months. It can even take up to a year…everything is a process…[which] makes you vulnerable*” (WCAU2).

“It's a story. Actually, it is a fight” (RAFU2).

### Theme 2: Purpose of a Lower Limb Prosthetic Guideline

**A.** Guiding clinical prescription responsive to user needs*:* A prosthesis must support individual function within a specific context and life role requirements. Users need their prosthesis to enable them to participate in activities meaningful to them. Therefore, prosthetic guidelines must ensure “*patients get the appropriate device, most clinically accurate for their diagnosis*” (KI4) and “*activity level*” (KI7). Prosthetic component prescription should “*not be a blanket approach*” (KI3) and not be driven by cost containment.

“*Reintegration vocationally, into the community, into family, is the one measure which any cost containment initiative should be able to measure*” (KI1).

Keeping in mind that “*employment and vocational needs that are unique*” (KI1), persons with lower limb amputations “*might not be able to return to work and live the fullest life because they are being kept back by what they are getting from their medical aid, government institution or workmans compensation*” (KI7).

Users concurred that they did not always get the most appropriate components to support their functioning.

“*I want a knee that can squat, that can run. I am still young, I want to run…I want to feel safe, because I used to fall at the mall… I just stood up and pretend like I'm okay, but when I get home, I feel like ohhh man*” (WCAU4).

Since the same person has different mobility needs due to different lifestyle requirements, a second prosthesis with components that support specific activities such as sport should be considered. Funders frequently do not make provision for a secondary prosthesis. Thus, persons with lower limb amputations “*use the prosthesis [for activities it was not meant] and suffer the consequences later*” (KI3) or the CPO “*try create that one shoe fits all hybridized prosthesis which might not be perfect for their daily ambulation, but at least allow them to attain some form of higher activity with regards to sporting events*” (KI3). A secondary prosthesis is also important when the primary prosthesis needs repairs. As explained by RAFU5, “*if I take the one in for repair, I use the other one*”.

**B.** Supporting adequate funding in a timely manner: Long waiting times were bemoaned by users and Key informants alike.

“*Patients' cases go on for years before they can get help*” (KI5). “*Quite a lengthy procedure*” (WCAU5). “*The process takes flippen [slang for very] long*” (RAFU2).

KI3 explained that “*there is one big challenge at the moment, and that would be funding.*” Although private funders fund high end prosthetic components, “*you might wait a while to be funded for it*” (KI7). The biggest concern was “*giving the correct prescription to the patient*” and “*at least ensuring to get reimbursed for the correct prescription made*” (KI3). This opinion was confirmed by KI7, “*funding is the main stumbling block between a patient being able to walk or being wheelchair bound or on crutches*”. “*Practitioners are often limited to give the patient the best possible solution due to limited funding which limits what the patient can achieve*” KI7. The RAF *“…will try to play out as long as they can before they pay*” (KI5).

Users agreed. “*The medical aid disappointed me…because they did not pay for the leg…If they could only fund the thing…They must at least fund a mechanical knee in full*” (CMSU3). In this instance the medical insurance paid 11% of the total cost of the prosthesis. The use of the prescribed minimum benefit when funding is supplied by medical insurance schemes was inconsistent and confusing. “*There is no standard approach*” (KI5) and “*It (PMB) is about interpretation and manipulation of interpretation…they try and make it as difficult as possible*” (KI3).

**C.** Equity, transparency, and fair re-imbursement: Guidelines can assist in ensuring fair, non-discriminatory treatment of all people with lower limb amputations. As indicated by KI1, “*Unified guidelines that speak to standardized care where all stakeholders can be held accountable should the guidelines not be adhered to…ethical conduct on the part of all concerned where the shift moves away from what prosthesis provides the most financial gain to what prosthesis provide the most in the patients activities of daily living*” (KI1).

Having standard guidelines for the provisioning of lower limb prostheses could be a win for all stakeholders involved.

“*Rules and regulations with the costing done correctly which will be saving money, patients getting better outcomes and suppliers getting better sales and reduce waste and abuse across the industry*” (KI3). “*Preventing fraud, over billing and unbundling*” (KI4).

Overprescription, related to prescribing more expensive rather than appropriate components, is a reality in the South African prosthetic industry “*Where prescription is written more for financial gain than to address the specific needs of the patient*” (KI5). RAFU6 concurred and explained an additional challenge related to overprescription. “*There are prosthetists who only prescribe the most expensive knees although the patient does not have the capability of using it. This makes it difficult for other persons with lower limb amputations to access such more expensive prosthetic knees when they actually need it*”.

The reimbursement of products historically *“were not (correctly) set up from the start where there was just no transparency. Any product could be listed at any price. It was a free for all and that is where the mistrust in the industry from the funders side*” (KI7) emanated from. It seemed as if unethical behaviour that involved financial gain were practiced by various stakeholders, not only prosthetists.

“*With RAF, it's the lawyers. Everyone is out there for money, and they forget about the patient, which becomes a struggle for the patient to get a prosthesis*” (KI5).

“*You can get a prosthesis, but you have to pay over funds into an account whereafter you will receive authorization. If you don't pay funds or don't put anything on the table, you get pushed to the side…the RAF has fired a lot of case managers, I think due to corruption*” (RAFU6).

“*There is an incredible amount of corruption unfortunately. Bribery is a big problem. Unfortunately, prosthetics are expensive commodities. At the end of the day, the guys do unethical, corrupt things to get the business and to the detriment of the patient*.” Patients get bribes “*in the form of cash incentive, fridges, cellphones that sort of things…. This undermines the profession, undermines the integrity of what we stand for and undermines the patient's rehabilitation outcome at the end of the day*” (KI3).

**D.** Multidisciplinary rehabilitation: The importance of receiving rehabilitation from a multi-disciplinary rehabilitation team was stressed.

“*Treating a patient holistically and understanding that the patients are full-on individuals that has different aspects to them*” (KI1) needs to be addressed by “*guidelines across the board which will unlock funding, treatment and rehabilitation*” (KI3).

“*There should be a dedicated program set out for any person with a lower limb amputation that covers enough sessions until that person is strengthened and reconditioned to probably where he was when he was still healthy and functional*” (CMSU5).

**E.** Case managers: Throughout the interviews it became evident that case managers can play an important role in accessing prosthetic care. The performance of the case manager mirrored the users' experience of accessing care. RAFU5 had the same “*case manager for longer than 10 years, it goes quick, I don't struggle*”. RAFU2 shared a different scenario. “*It took me a year to get a new case manager [after relocating to another province]. Their services were really bad*”. This caused delayed access to prosthetic care and RAFU2 personally funded the cost of prosthetic consumables for “*more than two years*”. Key informants also felt that RAF case managers might in some instances not fulfil their roles as required. “*Case managers are often really frustrating, some of them are really not good. Files get lost and patients are not contacted*” (KI3). KI7 stated, “*They don't answer phones. There is just no leadership and pride in what they are doing*”.

**F.** Information sharing: KI1 recommended “*Full-scale awareness to claimants to know what they qualify for according to the guidelines and to know what benefits are due to them…increase level of awareness, information and the entitlement to quality medical care*” (KI1).

WCAU5 indicated guideline should include information on:

- “*Best way”* to go about accessing prosthetic care.
- “*Time periods”* for when new components, sockets and consumables like liners, prosthetic socks, prosthetic foot covers etc. can be accessed.
- “*What is out there*” in terms of components for possible prescription.

**G.** A list of preferred practitioners: A guideline can also provide a list of preferred practitioners who provide high quality prosthetic care. Most people with a lower limb amputation have no reference point on how to identify a good CPO. “*You start to find your way through to somebody…and you find oh no, he is not good anymore. So, you go for a second opinion and somebody even better or probably even worse*” (WCAU5). CPOs play an intricate role in the provisioning of prostheses and the experience of persons using a lower limb prosthesis. “*This is somebody you need to be able to go on a journey with because that journey is probably going to be for the rest of your life*” (CMSU5). “*Get yourself a good prosthetist…it is a big deal*” (RAFU2).

### Theme 3: Guideline Development Requirements

**H.** Driven by an independent entity: Guidelines should “*try to find common ground and alignment of different interests*” (KI2). Thus, an independent body should be established to provide a “*neutral platform to engage*” (KI1). The entity should “*have no favoritism towards provider or funder and develop a guideline that shows fairness towards all parties, but most importantly showing value towards the patient*” (KI4).

**I.** Collaboration and communication: KI3 argued that successful guideline development should be an “*industry collaboration*” (KI3) following “*a multi-faceted approach*” (KI3) with “*a whole team*” (KI3). A “*multidisciplinary*” (KI6) and “*multi-stakeholder approach is important*” (KI1). This group of “*role players need to come in one room and develop something that will work best for the patients and for everyone else in the value chain*” (KI5) with the “*more stakeholders the better*” (KI6) where all “*agree on common good*” (KI2). Stakeholders include funders, service providers and users. “*The funder cannot do it without the service provider, and the service provider cannot do it without the funder*” (KI1). Academia was also seen as a “*major stakeholder*” (KI2) as they can do research on “*cost effectiveness, sourcing of materials to manufacture prosthetics and provide training*” (K12).

**J.** Use of available evidence: Key informants agreed that it was not necessary to “*reinvent the wheel*” (KI3) as “*cross referencing what we want to do and what is done internationally*” (KI3) can be done as “*international literature and experiences, drives a lot of evidence*” (KI1). Local guideline formation can “*borrow knowledge and expertise*” (KI1) from international counter parts where there “*is a strong drive on evidence-based medicine*” (KI1). The Unites States of America and Australia were identified to “*have similar situations to the South African private healthcare environment*” (KI4) and have “*guidelines which can be adjusted to fit the South African context*” (KI6). “*The International Society of Prosthetics and Orthotics which have good recommendations*” (KI6) can also be approached for guidance.

## DISCUSSION

Although the three cases were guided by different acts, they have the same mandate to fulfil - provisioning of lower limb prostheses. Thus, it is not surprising that findings were similar and overlapping. Findings support the development of an evidence-based guideline for provisioning of lower limb prosthetics in the South African private sector that can guide equitable, fair service delivery and provide clarity on treatment pathways, available components and prescription criteria as summarised in **Table 6**.

**Table 6:** Summary of study findings.

**Finding:** There is a need for developing a uniformed evidence-based guideline for the provisioning of lower limb prostheses in the South Africa private healthcare sector utilizing the AGREE II tool (main framework) and Delphi technique (for consensus purposes).	
**Addressing:** ▪ Equitable service delivery. ▪ Enhance and promoting ethical practice and industry transparency. ▪ Fair reimbursement. ▪ Prohibiting over-prescription and bribery. ▪ Encouraging economic activity of lower limb prosthetic users. ▪ Incorporating multiple stakeholder involvement and processes to increase guideline development rigour.	**Providing clarity on:** ▪ Treatment pathway for lower limb prosthetic users to follow. ▪ Available prosthetic components for prescription. ▪ Criteria for a new prosthesis and socket refits. ▪ Prescription on secondary prosthesis, repairs and maintenance of devices, sport and recreational prostheses. ▪ Rehabilitation treatment pathways.

Based on AGREE II findings current RAF guidelines cannot be recommended for use in the provision of lower limb prostheses while WCA and CMS guidelines can be recommended for use with modifications.^28^ However, acts and government gazette publications have a more overarching purpose than providing service delivery guidelines. Therefore, the low scores of the documents that were appraised with the AGREE II were hardly surprising. However, the low scores made it clear that these documents could not fulfil the role of a prosthetic service delivery guideline. The interviews confirmed this, and the data identified inconsistencies in products provided, uncertainty of processes, long waiting times and insufficient funding of products that point to a need for service delivery guidelines. Previous work suggested that guidelines can address these challenges.^8,12,32^

In South Africa resources are constrained and idealistic prescription to provide all lower limb prosthetic users with everything is not feasible. Thus, a guideline should weigh costs against function. However, as far as possible every user should be supplied with components which can best support their physical, vocational, recreational, and environmental requirements.^5,12^ The guideline should also provide guidance regarding the funding of a second prosthesis. In healthcare systems that fund secondary prostheses, provision of secondary prostheses are more prevalent among younger, transtibial prosthetic users with high mobility levels, who had non-dysvascular amputations.^33^ The provisioning of sport and recreational prostheses are cyclic in nature as a lack of an activity-specific prosthesis leads to lack of participation and lack of participation leads to inadequate justification for an activity-specific prosthesis.^32^

Rejecting applications without providing sound reasons or applications taking an inordinately long time to fund are determinantal to users' psychosocial experiences,^7,9,34^ quality of Life^34^ and economic activity.^5,35^ For children specifically, developmental milestones must be met to ensure optimal ongoing physical and emotional development.36 Not replacing prosthetic limbs timeously to accommodate growth and development needs can cause irreparable harm.^36^

Users described being unsure of processes and dependent on CPOs to secure funding. The level of support needed from CPOs might have been necessitated through unclear procedures, bureaucratic processes, stalling techniques, and red tape. A guideline written in plain language can help to facilitate user knowledge^8^ and thus user centered care.^37^ Unfortunately, dependence on CPO's increases the administration burden of CPO practices, the size of the administrative staff complements, and the cost of supplying a prosthesis.

Case managers can reduce the administrative burden on CPOs. They can guide the processes, provide a point of contact, provide feedback, assess user needs and much more. However, the funder as employer should ensure that case managers provide high-quality services and follow the correct procedures. Over and above guidelines, this can be guided by Standard Operating Procedures and disciplinary action.

A guideline should be in line with the study findings and AGREE II domains.^27^ The domains must be clearly identifiable throughout the guideline to ensure that the objectives, the issue dealt with (i.e. prosthetic service provision) and the target population are clearly described. All relevant stakeholders (including the target population) must be involved in the development of such a guideline. Evidence used to develop the guideline must be rigorous and used appropriately. The recommendations must be presented clearly and without ambiguity. Different pathways and possible deviation in some cases must be presented. The guideline must present strategies that can help its implementation as well as facilitators and barriers to implementation, including resource allocation and monitoring strategies. Competing interests of stakeholders and the view of the funder must not influence the guidelines. As indicated by participants and in the background, guidelines have been developed in other settings (mostly high-income countries).^8,12,32^ Information from these can be used to assist the development of guidelines in the South African private sector. However, caution must be used as the contexts differ and Sadeghi-Demneh et al**15** found that none of the guidelines currently in use internationally have been researched to determine their impact on service delivery.

### Limitations

The low numbers of CPOs and rehabilitation team members as participants could have limited a broader understanding of the available guidelines available for rehabilitating and providing prostheses for individuals with lower limb amputation. As the study was novel, no existing validated interview schedules were available and had to be self-formulated by the authors.

## CONCLUSION

The study highlighted the need for evidence-based guidelines to guide prosthetic service delivery in the South African private sector. Guidelines rather than protocols are recommended because guidelines are flexible and allow clinical judgement and adaptation to individual user needs. Guideline development must be driven by an independent body trusted by all stakeholders and follow a patient centric, transparent, equitable approach involving multiple stakeholders. The AGREE II tool and Delphi technique (where consensus has not been reached) can be used to facilitate the development process.

Further research should be conducted to guide the formation of a standardised lower limb prosthetic guideline facilitated by an independent body (consisting of multi-stakeholders in the industry) which provides the opportunity for adoption by all three cases studied. A method of reimbursement calculation for professional fees and prosthetic components should also be further explored.

## DECLARATION OF CONFLICTING INTERESTS

- **Bennie Theron**: Employee of Ossur South Africa.
- **Surona Visagie:** None.

## AUTHORS CONTRIBUTION

- **Bennie Theron:** Research design; Conceptualizing of the study; Data collection; Analysis and interpretation of the data; First draft of the manuscript; Manuscript finalization.
- **Surona Visagie:** Research design; Conceptualizing of the study; Analysis and interpretation of the data; Feedback on drafts of the manuscript.

## SOURCES OF SUPPORT

None.
